# Supplementary material for: Whether academics’ job performance makes a difference to burnout and the effect of psychological counselling—comparison of four types of performers
Source: PLoS One. 2024 Jun 14;19(6):e0305493. doi: 10.1371/journal.pone.0305493 (PMC11178174; doi:10.1371/journal.pone.0305493)
Supplement: S1 Table — (PDF) [file pone.0305493.s001.pdf]

S1 Table. Data for Table 5: Multiple regression analysis

Source	SS	df	MS			
				Number of obs	=	697
				F(6, 690)	=	129.33
Model	529.240162	6	88.2066937	Prob > F	=	0.0000
Residual	470.613496	690	.682048545	R-squared	=	0.5293
				Adj R-squared	=	0.5252
Total	999.853659	696	1.43657135	Root MSE	=	.82586

burnout	Coefficient	Std. err.	t	P>|t|	[95% conf.	interval]
gender	-.0354611	.0628534	-0.56	0.573	-.1588679	.0879457
maritals	.0603734	.0628224	0.96	0.337	-.0629725	.1837193
age	.0051701	.0285235	0.18	0.856	-.0508332	.0611734
adminposition	-.0447697	.0628537	-0.71	0.477	-.1681771	.0786377
workexp	-.0328637	.0158371	-2.08	0.038	-.0639584	-.0017689
kpi	-.0550108	.0019841	-27.73	0.000	-.0589065	-.0511152
_cons	5.545749	.1716906	32.30	0.000	5.208651	5.882848
