# Supplementary material for: Whether academics’ job performance makes a difference to burnout and the effect of psychological counselling—comparison of four types of performers
Source: PLoS One. 2024 Jun 14;19(6):e0305493. doi: 10.1371/journal.pone.0305493 (PMC11178174; doi:10.1371/journal.pone.0305493)
Supplement: S2 Table — (PDF) [file pone.0305493.s002.pdf]

S2 Table. Data for Table 6: Group linear regression analysis

| Source | SS | df | MS | | | |
|---|---|---|---|---|---|---|
| Model | 7.58030763 | 6 | 1.26338461 | Number of obs | = | 143 |
| Residual | 40.9371749 | 136 | .301008639 | F(6, 136) | = | 4.20 |
| | | | | Prob > F | = | 0.0007 |
| Total | 48.5174825 | 142 | .341672412 | R-squared | = | 0.1562 |
| | | | | Adj R-squared | = | 0.1190 |
| | | | | Root MSE | = | .54864 |

| burnout | Coefficient | Std. err. | t | P>\|t\| | [95% conf. | interval] |
|---|---|---|---|---|---|---|
| gender | -.006112 | .093447 | -0.07 | 0.948 | -.1909091 | .1786852 |
| maritals | .0017301 | .0937149 | 0.02 | 0.985 | -.1835968 | .1870571 |
| age | .0324222 | .0416208 | 0.78 | 0.437 | -.0498854 | .1147297 |
| adminposition | -.2850114 | .0930945 | -3.06 | 0.003 | -.4691114 | -.1009113 |
| workexp | -.0225826 | .0240781 | -0.94 | 0.350 | -.0701985 | .0250334 |
| kpi | -.0562722 | .0153619 | -3.66 | 0.000 | -.0866513 | -.0258932 |
| _cons | 5.80982 | 1.455034 | 3.99 | 0.000 | 2.932402 | 8.687239 |

| Source | SS | df | MS | | | |
|---|---|---|---|---|---|---|
| Model | 218.301549 | 6 | 36.3835915 | Number of obs | = | 282 |
| Residual | 104.506962 | 275 | .380025316 | F(6, 275) | = | 95.74 |
| | | | | Prob > F | = | 0.0000 |
| Total | 322.808511 | 281 | 1.14878474 | R-squared | = | 0.6763 |
| | | | | Adj R-squared | = | 0.6692 |
| | | | | Root MSE | = | .61646 |

| burnout | Coefficient | Std. err. | t | P>\|t\| | [95% conf. | interval] |
|---|---|---|---|---|---|---|
| gender | .0890534 | .074994 | 1.19 | 0.236 | -.0585819 | .2366888 |
| maritals | -.0433805 | .0749385 | -0.58 | 0.563 | -.1909066 | .1041456 |
| age | -.0334338 | .0330567 | -1.01 | 0.313 | -.0985102 | .0316426 |
| adminposition | -.0156137 | .0738879 | -0.21 | 0.833 | -.1610715 | .1298441 |
| workexp | -.0201404 | .0182727 | -1.10 | 0.271 | -.0561125 | .0158317 |
| kpi | -.1574109 | .0066896 | -23.53 | 0.000 | -.1705802 | -.1442416 |
| _cons | 13.70186 | .5467171 | 25.06 | 0.000 | 12.62558 | 14.77814 |

| Source | SS | df | MS | | |
|---|---|---|---|---|---|
| Model | 101.330806 | 6 | 16.8884677 | Number of obs = | 141 |
| Residual | 53.4067826 | 134 | .398558079 | F(6, 134) = | 42.37 |
| | | | | Prob > F = | 0.0000 |
| Total | 154.737589 | 140 | 1.10526849 | R-squared = | 0.6549 |
| | | | | Adj R-squared = | 0.6394 |
| | | | | Root MSE = | .63131 |

| burnout | Coefficient | Std. err. | t | P>\|t\| | [95% conf. | interval] |
|---|---|---|---|---|---|---|
| gender | -.1922213 | .1107553 | -1.74 | 0.085 | -.411276 | .0268334 |
| maritals | -.0059956 | .1097216 | -0.05 | 0.957 | -.2230058 | .2110147 |
| age | .0118491 | .0520906 | 0.23 | 0.820 | -.091177 | .1148752 |
| adminposition | -.0507703 | .1111618 | -0.46 | 0.649 | -.2706289 | .1690883 |
| workexp | -.0229711 | .0265171 | -0.87 | 0.388 | -.0754174 | .0294751 |
| kpi | -.2956434 | .018888 | -15.65 | 0.000 | -.3330006 | -.2582861 |
| _cons | 21.30722 | 1.219766 | 17.47 | 0.000 | 18.89473 | 23.7197 |

| Source | SS | df | MS | | |
|---|---|---|---|---|---|
| Model | 49.3560165 | 6 | 8.22600275 | Number of obs = | 131 |
| Residual | 54.3081057 | 124 | .437968594 | F(6, 124) = | 18.78 |
| | | | | Prob > F = | 0.0000 |
| Total | 103.664122 | 130 | .797416324 | R-squared = | 0.4761 |
| | | | | Adj R-squared = | 0.4508 |
| | | | | Root MSE = | .66179 |

| burnout | Coefficient | Std. err. | t | P>\|t\| | [95% conf. | interval] |
|---|---|---|---|---|---|---|
| gender | -.2083586 | .1188468 | -1.75 | 0.082 | -.4435898 | .0268726 |
| maritals | .0342631 | .1181162 | 0.29 | 0.772 | -.199522 | .2680482 |
| age | .0528995 | .0561597 | 0.94 | 0.348 | -.0582563 | .1640552 |
| adminposition | .0116881 | .1183718 | 0.10 | 0.922 | -.2226029 | .2459791 |
| workexp | -.0633802 | .0317408 | -2.00 | 0.048 | -.1262042 | -.0005562 |
| kpi | -.0995049 | .0102315 | -9.73 | 0.000 | -.1197559 | -.0792539 |
| _cons | 7.380174 | .5672587 | 13.01 | 0.000 | 6.25741 | 8.502938 |
