# Supplementary material for: Whether academics’ job performance makes a difference to burnout and the effect of psychological counselling—comparison of four types of performers
Source: PLoS One. 2024 Jun 14;19(6):e0305493. doi: 10.1371/journal.pone.0305493 (PMC11178174; doi:10.1371/journal.pone.0305493)
Supplement: S3 Table — (PDF) [file pone.0305493.s003.pdf]

S3 Table. Data for Figure 3: Job performance group comparison among academics over time (2019 to 2023)

A(X)	B(Y)	C(Y)	D(Y)	E(Y)
Year	Non perfor	Low perfor	Average pe	High perfo
2019	2.496	2.132	0.986	0.667
2020	2.397	2.261	0.993	0.532
2021	2.461	2.315	1.044	0.613
2022	2.537	2.156	1.03	0.64
2023	2.237	2.227	1.106	0.35
