# Supplementary material for: Whether academics’ job performance makes a difference to burnout and the effect of psychological counselling—comparison of four types of performers
Source: PLoS One. 2024 Jun 14;19(6):e0305493. doi: 10.1371/journal.pone.0305493 (PMC11178174; doi:10.1371/journal.pone.0305493)
Supplement: S4 Table — (PDF) [file pone.0305493.s004.pdf]

S4 Table. Data for Table 7: Hierarchical regression analysis

Source	SS	df	MS			
Model	6.04812035	5	1.20962407	Number of obs	=	457
Residual	286.332624	451	.634883866	F(5, 451)	=	1.91
				Prob > F	=	0.0921
Total	292.380744	456	.641185842	R-squared	=	0.0207
				Adj R-squared	=	0.0098
				Root MSE	=	.7968

burnout	Coefficient	Std. err.	t	P>|t|	[95% conf.	interval]
gender	-.0510408	.075329	-0.68	0.498	-.1990801	.0969986
maritals	-.1088789	.0746373	-1.46	0.145	-.2555589	.0378012
age	.0592008	.0348676	1.70	0.090	-.0093222	.1277238
adminposition	.0596826	.0746809	0.80	0.425	-.0870831	.2064483
workexp	-.0331487	.0189165	-1.75	0.080	-.070324	.0040267
_cons	2.173097	.1081755	20.09	0.000	1.960507	2.385688

Source	SS	df	MS			
Model	77.5176455	6	12.9196076	Number of obs	=	457
Residual	214.863098	450	.477473552	F(6, 450)	=	27.06
				Prob > F	=	0.0000
Total	292.380744	456	.641185842	R-squared	=	0.2651
				Adj R-squared	=	0.2553
				Root MSE	=	.69099

burnout	Coefficient	Std. err.	t	P>|t|	[95% conf.	interval]
gender	-.0665006	.0653388	-1.02	0.309	-.1949076	.0619064
maritals	-.0347074	.06501	-0.53	0.594	-.1624683	.0930534
age	.0306768	.0303275	1.01	0.312	-.0289243	.0902778
adminposition	-.0187468	.065081	-0.29	0.773	-.1466471	.1091536
workexp	-.0334517	.0164047	-2.04	0.042	-.065691	-.0012124
kpi	-.0289409	.0023655	-12.23	0.000	-.0335897	-.0242921
_cons	4.153942	.1871213	22.20	0.000	3.786202	4.521682

Source	SS	df	MS			
				Number of obs	=	457
				F(6, 450)	=	2.28
Model	8.63113314	6	1.43852219	Prob > F	=	0.0352
Residual	283.749611	450	.630554691	R-squared	=	0.0295
				Adj R-squared	=	0.0166
Total	292.380744	456	.641185842	Root MSE	=	.79407

burnout	Coefficient	Std. err.	t	P>|t|	[95% conf.	interval]
gender	-.0494718	.0750757	-0.66	0.510	-.1970143	.0980708
maritals	-.1071157	.0743875	-1.44	0.151	-.2533058	.0390743
age	.0591506	.0347485	1.70	0.089	-.0091389	.12744
adminposition	.0527181	.0745053	0.71	0.480	-.0937035	.1991397
workexp	-.0320074	.0188603	-1.70	0.090	-.0690725	.0050578
counseling	-.1506495	.074433	-2.02	0.044	-.296929	-.00437
_cons	2.248858	.1141197	19.71	0.000	2.024585	2.473132

Source	SS	df	MS			
				Number of obs	=	457
				F(8, 448)	=	21.91
Model	82.2231275	8	10.2778909	Prob > F	=	0.0000
Residual	210.157616	448	.469101822	R-squared	=	0.2812
				Adj R-squared	=	0.2684
Total	292.380744	456	.641185842	Root MSE	=	.68491

burnout	Coefficient	Std. err.	t	P>|t|	[95% conf.	interval]
gender	-.0450319	.0651256	-0.69	0.490	-.1730216	.0829578
maritals	-.0403192	.064464	-0.63	0.532	-.1670086	.0863703
age	.0299752	.0300631	1.00	0.319	-.0291069	.0890574
adminposition	-.0248077	.0645603	-0.38	0.701	-.1516865	.102071
workexp	-.030248	.0162933	-1.86	0.064	-.0622687	.0017727
kpi	-.0215938	.0033175	-6.51	0.000	-.0281135	-.0150741
counseling	.8973938	.3191852	2.81	0.005	.2701076	1.52468
kpic	-.0143448	.0047053	-3.05	0.002	-.023592	-.0050976
_cons	3.693179	.2419203	15.27	0.000	3.21774	4.168619
