# Supplementary material for: Whether academics’ job performance makes a difference to burnout and the effect of psychological counselling—comparison of four types of performers
Source: PLoS One. 2024 Jun 14;19(6):e0305493. doi: 10.1371/journal.pone.0305493 (PMC11178174; doi:10.1371/journal.pone.0305493)
Supplement: S5 Table — (PDF) [file pone.0305493.s005.pdf]

S5 Table. Data for Table 8: Group regression analysis

Source	SS	df	MS			
Model	4.22942321	8	.528677901	Number of obs	=	87
Residual	18.7590825	78	.240501058	F(8, 78)	=	2.20
Total	22.9885057	86	.267308206	Prob > F	=	0.0363
				R-squared	=	0.1840
				Adj R-squared	=	0.1003
				Root MSE	=	.49041

burnout	Coefficient	Std. err.	t	P>|t|	[95% conf.	interval]
gender	.1438228	.1117949	1.29	0.202	-.0787436	.3663893
maritals	.0510522	.1092687	0.47	0.642	-.166485	.2685895
age	-.0486547	.0491677	-0.99	0.325	-.1465402	.0492308
adminposition	-.1615668	.1100293	-1.47	0.146	-.3806184	.0574847
workexp	-.0343772	.0290203	-1.18	0.240	-.0921522	.0233979
kpi	-.0612899	.0178187	-3.44	0.001	-.0967643	-.0258155
counselingfreq	-.1172704	.4934033	-0.24	0.813	-1.099561	.8650202
kpicounselingfreq	.005373	.0234536	0.23	0.819	-.0413196	.0520656
_cons	6.455086	1.956691	3.30	0.001	2.559614	10.35056

Source	SS	df	MS			
Model	44.0101798	8	5.50127248	Number of obs	=	53
Residual	2.29170695	44	.052084249	F(8, 44)	=	105.62
Total	46.3018868	52	.8904209	Prob > F	=	0.0000
				R-squared	=	0.9505
				Adj R-squared	=	0.9415
				Root MSE	=	.22822

burnout	Coefficient	Std. err.	t	P>|t|	[95% conf.	interval]
gender	-.1341415	.0646663	-2.07	0.044	-.2644679	-.0038151
maritals	-.0766079	.0649196	-1.18	0.244	-.2074446	.0542289
age	.0138392	.0353214	0.39	0.697	-.0573464	.0850248
adminposition	-.0397661	.0655918	-0.61	0.547	-.1719576	.0924254
workexp	-.0181621	.0174482	-1.04	0.304	-.0533267	.0170025
kpi	-.0980071	.0110322	-8.88	0.000	-.1202411	-.0757731
counselingfreq	-1.575033	.2263065	-6.96	0.000	-2.031123	-1.118942
kpicounselingfreq	-.0364347	.0110845	-3.29	0.002	-.0587741	-.0140953
_cons	10.03907	.9700156	10.35	0.000	8.084129	11.99401

Source	SS	df	MS			
Model	101.491717	8	12.6864647	Number of obs	=	151
Residual	41.4486799	142	.291892112	F(8, 142)	=	43.46
				Prob > F	=	0.0000
Total	142.940397	150	.952935982	R-squared	=	0.7100
				Adj R-squared	=	0.6937
				Root MSE	=	.54027

burnout	Coefficient	Std. err.	t	P>|t|	[95% conf.	interval]
gender	.1147485	.0916024	1.25	0.212	-.0663322	.2958292
maritals	-.0432203	.0913739	-0.47	0.637	-.2238494	.1374087
age	-.0439438	.0406814	-1.08	0.282	-.1243633	.0364757
adminposition	-.0120544	.0900495	-0.13	0.894	-.1900652	.1659563
workexp	-.003884	.0226952	-0.17	0.864	-.0487482	.0409802
kpi	-.1422531	.0081905	-17.37	0.000	-.1584442	-.126062
counselingfreq	.0329084	.0925251	0.36	0.723	-.1499964	.2158131
kpicounselingfreq	.0046894	.0101446	0.46	0.645	-.0153645	.0247433
_cons	12.3564	.7124478	17.34	0.000	10.94803	13.76478

Source	SS	df	MS			
Model	69.8208	8	8.7276	Number of obs	=	82
Residual	28.4231024	73	.389357568	F(8, 73)	=	22.42
				Prob > F	=	0.0000
Total	98.2439024	81	1.21288768	R-squared	=	0.7107
				Adj R-squared	=	0.6790
				Root MSE	=	.62399

burnout	Coefficient	Std. err.	t	P>|t|	[95% conf.	interval]
gender	-.0539166	.1498159	-0.36	0.720	-.3524992	.244666
maritals	.0405342	.1490242	0.27	0.786	-.2564706	.337539
age	.1037925	.0696352	1.49	0.140	-.0349903	.2425752
adminposition	-.0505318	.1529263	-0.33	0.742	-.3553135	.25425
workexp	-.0287509	.0355146	-0.81	0.421	-.0995315	.0420297
kpi	-.2721001	.0374468	-7.27	0.000	-.3467315	-.1974687
counselingfreq	-2.106537	.7182367	-2.93	0.004	-3.537981	-.6750932
kpicounselingfreq	-.3487123	.1035226	-3.37	0.001	-.5550325	-.1423921
_cons	23.39897	1.776305	13.17	0.000	19.8588	26.93914
