# Supplementary material for: Whether academics’ job performance makes a difference to burnout and the effect of psychological counselling—comparison of four types of performers
Source: PLoS One. 2024 Jun 14;19(6):e0305493. doi: 10.1371/journal.pone.0305493 (PMC11178174; doi:10.1371/journal.pone.0305493)
Supplement: S6 Table — (PDF) [file pone.0305493.s006.pdf]

S6 Table. Data for Figure 4: Burnout comparison based on the role of psychological counselling (2019 to 2023)

A(X)	B(Y)	C(Y)
Year	Non-psycho	Psychologi
2019	1.5	1.422
2020	1.553	1.43
2021	1.481	1.467
2022	1.517	1.477
2023	1.515	1.282
