# Supplementary material for: Whether academics’ job performance makes a difference to burnout and the effect of psychological counselling—comparison of four types of performers
Source: PLoS One. 2024 Jun 14;19(6):e0305493. doi: 10.1371/journal.pone.0305493 (PMC11178174; doi:10.1371/journal.pone.0305493)
Supplement: S7 Table — (PDF) [file pone.0305493.s007.pdf]

S7 Table. Data for Figure 5: KPI performance comparison based on the frequency of psychological counselling (2019 to 2023)

A(X)	B(Y)	C(Y)	D(Y)	E
Year	Monthly Se	Bi-Weekly	Weekly Ses	
2019	74.339	74.162	73.655	
2020	73.906	74.971	74.098	
2021	74.407	74.413	73.89	
2022	73.563	72.739	74.261	
2023	73.92	75.894	76.67	
