# Supplementary material for: Whether academics’ job performance makes a difference to burnout and the effect of psychological counselling—comparison of four types of performers
Source: PLoS One. 2024 Jun 14;19(6):e0305493. doi: 10.1371/journal.pone.0305493 (PMC11178174; doi:10.1371/journal.pone.0305493)
Supplement: Attachment — Submitted filename: The reviwer comments.docx [file pone.0305493.s008.docx]

**Title: Whether academics’ job performance makes a difference to burnout and the effect of psychological counselling—comparison of four types of performers**

**Version Date:** 13 April 2024.

**Reviewer:** Heba Moussa.

**I have assessed your manuscript and would like to kindly request a clarification:**

**Abstract**

- **Keywords:** Please consider visiting Mesh to select your keywords. using is very helpful in terms of increasing your article visibility & citation chance! Please Visit Their Website: <https://www.ncbi.nlm.nih.gov/mesh/\>

**You need to organize the manuscript more properly. There are several recurring paragraphs, such as those related to the history of research into the causes of burnout, which makes the research complicated to read and confusing.**

**Background**

- **The study aim:** it was very long and make confused. The introduction for the aim section is not necessary. Please mention the study aim directly.
- Fig 2 in page 7 don’t clarify the psychological counseling role as a mediator as you mentioned in the research hypothesize in page 9. Please clarify in the figure.
- I suggest to merge the Research Context section in page 11 with the literature review with avoid repetition. It is too long and hard to read.

**Methods**

- The study design is not clear. What type of the quantitative method was used? and the used design should be supported by a cited materials that justifies using it.
- Some main missing points: a brief description of the study setting and Chinese universities, population, target universities. I suggest adding separate paragraph for the study setting description.
- The study sample section needs to organize. You mentioned in the beginning you used (a multi-stage sampling approach). Then, you mentioned in the second paragraph (a stratified sampling technique). I suggest it need to arranged in the beginning of the section as, **a stratified sampling technique was used by** **divides the population into smaller groups (strata) based on shared characteristics of the target individual**.
- Please clarify the used sample size formula.
- Sample size and grouping were clear enough and organized in table 1.
- Inclusion and exclusion criteria should be mentioned in the sample section not in data collection section.
- The tools of data collection not mentioned. Please describe the used tools in details, clarify if developed by the researcher or adapted from original references, and identify the scoring system used.

**Validity**

I have read the entire methods section. However, I could not find any details about tools validity. Please describe tools validity in separate section entitled (tools validity).

**Reliability**

It not mentioned. Please explain the study tools’ reliability.

**Field of work**

Please explain the method and time of questionnaires distribution.

**Statical analysis**

It is clear in table 4.

**Results and discussion**

I see that the discussion was answered the research hypothesis.
