# Supplementary material for: Whether academics’ job performance makes a difference to burnout and the effect of psychological counselling—comparison of four types of performers
Source: PLoS One. 2024 Jun 14;19(6):e0305493. doi: 10.1371/journal.pone.0305493 (PMC11178174; doi:10.1371/journal.pone.0305493)
Supplement: Attachment — Submitted filename: PLOS_ONE - PONE-D-24-07762 - Review.docx [file pone.0305493.s009.docx]

# Weather Academics’ Job Performance Makes a Difference to Burnout and the Effect of Psychological Counselling-Comparison of Four Types of Performers.

***Comments to the Author**

## 1. Is the manuscript technically sound, and do the data support the conclusions?

*The manuscript must describe a technically sound piece of scientific research with data that supports the conclusions. Experiments must have been conducted rigorously, with appropriate controls, replication, and sample sizes. The conclusions must be drawn appropriately.*

The manuscript demonstrates technical soundness through rigorous statistical analysis, systematic methodology, and appropriate data-conclusion alignment, supporting the validity of the research findings.

The research paper conducted group regression analysis among four performance groups to determine the moderating effect of psychological counseling, ensuring a rigorous statistical approach. The study utilized multiple regression analysis to explore the relationship between job performance and burnout, demonstrating a methodical data analysis process.

Additionally, the manuscript's methodology involved a quantitative approach, ensuring a structured and systematic research design. The data collection procedures were carefully explained, with anonymity measures in place to protect participants' privacy and ensure research integrity. Also, the conclusions drawn in the paper were based on the data presented, with a clear link established between job performance, burnout, and the moderating effect of psychological counseling.

## 2. Has the statistical analysis been performed appropriately and rigorously?

The statistical analysis in the research paper appears to have been performed appropriately and rigorously, utilizing various statistical techniques to investigate the research questions effectively.

The research paper employed group regression analysis among four performance groups to determine the moderating effect of psychological counseling, showcasing a robust statistical approach. Multiple regression analysis was utilized to investigate the relationship between academics' performance and job burnout, indicating a comprehensive statistical analysis methodology. The study justified the use of quantitative research to establish clear statistical outcomes and relationships between variables, highlighting the appropriateness of the statistical methods employed.

In addition, a multi-stage sampling approach was implemented to achieve a representative sample, ensuring the statistical analysis was conducted with careful consideration of sample heterogeneity and data accuracy. In turn, the statistical methods used in the research paper align with the study's objectives, providing a rigorous analysis framework to explore the dynamics between job performance, burnout, and the moderating effect of psychological counseling.

## 3. Have the authors made all data underlying the findings in their manuscript fully available?

*The PLOS Data policy requires authors to make all data underlying the findings described in their manuscript fully available without restriction, with rare exception (please refer to the Data Availability Statement in the manuscript PDF file). The data should be provided as part of the manuscript or its supporting information, or deposited to a public repository. For example, in addition to summary statistics, the data points behind means, medians and variance measures should be available. If there are restrictions on publicly sharing data—e.g. participant privacy or use of data from a third party—those must be specified.*

Authors have taken measures to maintain data confidentiality, collect comprehensive demographic information, and rely on reliable sources, ensuring the availability of essential data for the study.

Authors have ensured the anonymity of participants by assigning numerical codes sequentially and using pseudonyms for universities, maintaining confidentiality. Data on burnout and psychological counseling were directly obtained from the Mental Health Centre of sampled universities, ensuring reliable information for the study.

The collected demographic data includes gender, marital status, age, administrative position, and years of employment, providing a comprehensive dataset for analysis.

While the specific raw data points behind means, medians, and variance measures are not explicitly mentioned, the study's reliance on secondary data from universities' personnel departments enhances the reliability and availability of data for further analysis.

## 4. Is the manuscript presented in an intelligible fashion and written in standard English?

*PLOS ONE does not copyedit accepted manuscripts, so the language in submitted articles must be clear, correct, and unambiguous. Any typographical or grammatical errors should be corrected at revision, so please note any specific errors here.*

The manuscript is well-written, free from language errors, and presented in a manner that is easily understandable, meeting the standards of clarity and language proficiency expected in academic publications. It is presented in a clear and intelligible fashion, adhering to standard English conventions throughout the text. Authors have effectively communicated their research findings, hypotheses, and implications in a coherent manner, enhancing the readability and understanding of the study.
